# New Insights into Molecular Mechanisms Mediating Adaptation to Exercise; A Review Focusing on Mitochondrial Biogenesis, Mitochondrial Function, Mitophagy and Autophagy

**DOI:** 10.3390/cells10102639

**Published:** 2021-10-02

**Authors:** Fiona Louise Roberts, Greg Robert Markby

**Affiliations:** Nutrient and Metabolite Sensing, Novo Nordisk Foundation Center for Basic Metabolic Research, University of Copenhagen, 2200 Copenhagen, Denmark; fiona.roberts@sund.ku.dk

**Keywords:** exercise, molecular signaling, autophagy, mitophagy and mitochondrial biogenesis

## Abstract

Exercise itself is fundamental for good health, and when practiced regularly confers a myriad of metabolic benefits in a range of tissues. These benefits are mediated by a range of adaptive responses in a coordinated, multi-organ manner. The continued understanding of the molecular mechanisms of action which confer beneficial effects of exercise on the body will identify more specific pathways which can be manipulated by therapeutic intervention in order to prevent or treat various metabolism-associated diseases. This is particularly important as exercise is not an available option to all and so novel methods must be identified to confer the beneficial effects of exercise in a therapeutic manner. This review will focus on key emerging molecular mechanisms of mitochondrial biogenesis, autophagy and mitophagy in selected, highly metabolic tissues, describing their regulation and contribution to beneficial adaptations to exercise.

## 1. Introduction

**Figure 1:**
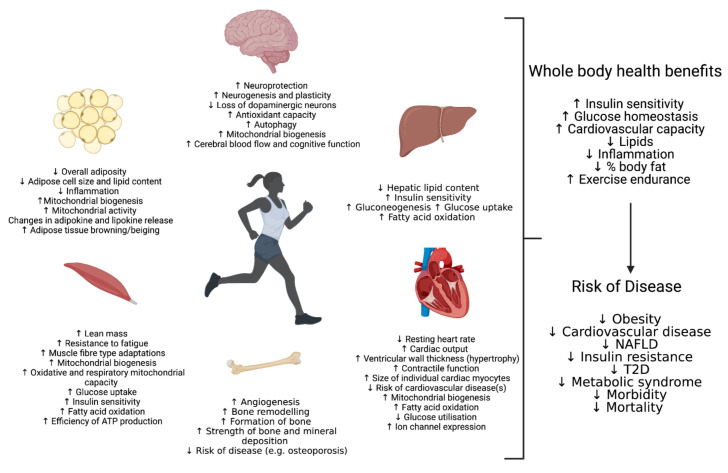
Physiological Adaptations in response to exercise stress in selected metabolic tissues.

Exercise is fundamental for maintaining good health over the course of a lifetime. Not only do regular bouts of exercise confer a myriad of metabolic benefits in a range of tissues, but also a single bout of acute exercise can begin to activate molecular pathways and adaptions important to health [1,2,3,4,5] (Figure 1). Indeed, physical fitness can be used as a quantitative predictor for overall mortality and can be used to measure an individual’s quality of life [6]. Conversely, the increase in sedentary lifestyle has been strongly associated with an increase in the incidence of obesity, cardiovascular disease, type 2 diabetes (T2D), psychological and neurological health conditions and other negative health effects [7,8,9,10]. However, despite the clear benefits to exercise, full comprehension of the underlying molecular mechanisms involved in the human bodies adaptive response to exercise remains to be determined. This is due to not only the complexity of the exercise response but also the number of variables that can affect how the body will adapt. Such variables include the duration, frequency, intensity and type of exercise (aerobic or resistance) as well as the heterogeneity within the population including factors such as age, diet, sex and race, further complicated by the identification of groups of high, low or even negative responders within the population and stratified subpopulations [5,11,12,13]. Ageing has a recognized effect on the capacity to respond to exercise, with evidence demonstrating negatively impacted capabilities in the elderly. This review will focus on mechanisms described in young cohorts, with a detailed assessment of the effect of ageing being beyond the scope of this discussion. Furthermore, it is important to consider that over-exercising can have detrimental effects on health and cellular homeostasis with a recent study indicating overtraining causes mitochondrial dysfunction and glucose intolerance [14]. It is also critical to note that exercise is not an available option to all individuals due to various health conditions. Given this, it is imperative that more is understood regarding molecular mechanisms controlling the adaptive response so that individuals can tailor their exercise regime to maximise the associated health benefits. Alternatively, the identification of critical pathways in the positive adaptation to exercise may identify potential therapeutic targets to enhance such pathways and thus improve physiology both in patients with and without the ability to perform exercises.

Research, in recent years, has been attempting to unravel the complex nature of exercise adaption with an ever-increasing repertoire of molecular tools and exercise models. The application of these advanced toolkits has been illuminating the central pathways involved. The use of retrospective studies, appropriate use of animal models and careful appraisal of translational scope, human meta-analysis and assessment of both short term and longer-term exercise has likewise been employed. This has identified a number of key pathways and signalling molecules that detect the metabolic and mechanical stresses induced during exercise and work to not only restore cellular homeostasis but also induce adaptive changes across the body to improve the response to these challenges in future. This includes energy and nutrient sensors such as AMP kinase (AMPK) and mechanistic target of rapamycin (mTOR), intracellular signalling pathways such as phosphatidylinositol 3-kinase-protein kinase B (PI3K-Akt) pathway and transcriptional regulators such as Peroxisome proliferator-activated receptor gamma coactivator 1-alpha (PGC-1α) and sirtuin 1 (SIRT1) to name but a few [5,15,16,17,18,19,20,21,22,23,24]. These signalling pathways alone can confer an extensive list of changes to cellular processes and adaptions both in specific tissues but also throughout the whole body through endocrine signalling pathways. It is outside the scope of this article to discuss all aspects of molecular signalling and adaptions involved in the response to exercise. Instead, this review focuses on the processes of macro-autophagy and the balance between mitochondrial biogenesis and targeted mitochondrial removal otherwise known as mitophagy. Emerging evidence points towards these processes facilitating multi-organ metabolically favourable exercise adaptations. We will give a contemporary discussion of the molecular mechanisms behind these processes in various metabolically important tissues (skeletal muscle, liver, cardiovascular and adipose) in response to exercise and highlight the beneficial metabolic outcomes they confer.

### Emerging Important Molecular Mechanisms in the Regulation of Exercise Adaptation:

To appreciate and understand these processes fully in the context of exercise response, we must first define and characterise each process. Autophagic turnover is the process by which cells clear defunct and dysfunctional organelles and cytosolic components to be recycled, it is broadly classified into three sub-groups; micro-, macro- and chaperone-mediated. Here, we will focus on the most widely studied of these groups, macro-autophagy (henceforth termed autophagy). Autophagy, briefly, begins with the formation of an autophagosome, a double-membrane vesicle that engulfs substrates, this then subsequently fuses with a lysosome, thus forming the autolysosome. The material trapped within undergoes lysosomal hydrolyses and is degraded and released to be recycled as amino acids [25]. A number of key genes (termed autophagy-related genes or ATGs) and proteins are required in the initiation and control of autophagy. This includes proteinssuch as Unc-51 autophagy activating kinase 1 (ULK1), which exists in a complex with FIP200, ATG13 and ATG101, and when dephosphorylated initiates the formation of the autophagosome through the phosphorylation of BECLIN-1 which itself exists in a complex [26,27,28]. The microtubule-associated protein 1 light chain 3 (LC3) is also critical for the formation of the autophagosome membrane [29,30]. The autophagy-specific gene (Atg)-4 cleaves pro-LC3 resulting in the mature form of LC3-I [29]. Later this LC3-I is conjugated to the autophagosome membrane by ATG7 and ATG3 and forms a lipidated conjugate referred to as LC3-II [29]. This process simultaneously closes the autophagosome whilst initiating the trafficking of the completed autophagosome to lysosomes for degradation [31,32]. Finally, proteins such as transcription factor EB (TFEB) and, to a certain extent, its related family member transcription factor binding To IGHM enhancer 3 (TFE3) plays a critical role in the biogenesis of lysosomes themselves acting as transcription factors that, when dephosphorylated, translocate to the nucleus to induce lysosomal genes [33,34]. Likewise, members of the Forkhead box O (FOXO) family of transcription factors, specifically FOXO3 and FOXO1, have also been connected to autophagy, with their translocation to the nucleus inducing ATGs including LC3 [35,36,37,38,39]. Interestingly, all these pathways have shown to be controlled or influenced by a number of the key regulator proteins involved in exercise and will be discussed in a tissue-specific manner later [35,38,39,40,41,42].

Autophagy can be non-specific, whereby an area of the cytoplasm is engulfed, or targeted through which the autophagosome is directed to engulf specific organelles or substrates for degradation. One example of this targeted approach, termed mitophagy, is the engulfment of predominantly defunct/dysfunctional mitochondria [43]. This process, alongside mitochondrial biogenesis (discussed later), is seen as essential to maintain a healthy and functioning cell, not only maintaining homeostasis in fully differentiated tissues basally but also playing a role in the differentiation process of different tissues as well as the adaption of tissues in response to different cellular stress [44,45]. Broadly speaking, mitophagy uses many of the same mechanisms observed in general autophagy but utilises certain proposed strategies to guide the autophagosome formation around the mitochondria, targeted for destruction [45,46]. The most well studied of these is the PTEN-induced kinase 1 (PINK1) and PARKIN mechanism for mitochondrial targeting. Briefly, whilst the mitochondria fully functioning PINK1 is translocated across the outer and then inner mitochondrial membranes before being cleaved by the protease preselin-associated rhomboid-like (PARL) [44,45,47,48]. The two subsequent fragments are then degraded, one in the mitochondria and the other translocates back out of the mitochondria and degraded in the cytoplasm, with this process happening in a constant and rapid fashion [45,47,48,49,50]. As the mitochondria age or become disordered, this process is disrupted resulting in an accumulation of PINK1 at the outer membrane of the mitochondria where it becomes autophosphorylated and interacts with PARKIN, an E3 ubiquitin ligase, initiating recruitment of autophagy machinery [49,51,52,53]. In addition to this mechanism, a number of other pathways have been associated with mitophagy induction including the BCL2/adenovirus E1B protein-interacting protein 3-like (BNIPL3) NIX pathway, the protein FUN14 domain containing 1 (FUNDC1), cardiolipin (CL), prohibitin 2 (PHB2), FK506-binding protein 8 (FKBP8), BCL2 Like 13 (BCL2L-13) and the autophagy and Beclin 1 regulator (AMBRA1)-containing complex of proteins [45,54,55,56,57,58,59,60,61]. The induction of mitophagy by these mechanisms is not always mutually exclusive, complicating the understanding of the regulation of this process. However, like general autophagy, a number of proteins implicated in exercise have been implicated in the control and induction of this pathway.

Whilst it is essential to clear dysfunctional mitochondria from the cell, it is likewise imperative that new and functioning mitochondria are created. Through the division of pre-existing mitochondria, via an auto replication mechanism, the number of mitochondria can increase; this process is termed mitochondrial biogenesis. The initial observations of this process was in comparing exercised and non-exercised muscle tissue fragments, first in birds and then in rodents where John Holloszy’s pioneering work stipulated that the increased mitochondrial electron transport observed in exercised muscle samples is likely due to an increase in mitochondrial biogenesis [62,63]. Regulation of mitochondrial biogenesis requires the coordination of both nuclear and mitochondrial encoded genes with the vast majority of these being encoded in the nucleus with only 13 proteins being encoded in the mitochondria [64,65,66]. Mitochondrial biogenesis being observed first in exercised muscle samples is perhaps unsurprising given the master regulator in this process PGC-1α, as previously mentioned, is highly regulated in response to exercise [15,16,65,67]. When PGC-1α is deacetylated and phosphorylated it becomes active inducing the transcription of a number of genes including the mitochondrial transcription factor A (TFAM) that directs both nuclear and mitochondrial gene expression by interacting with mitochondrial promoter DNA enhancing gene expression of mitochondrial genes [67,68]. Regulation of PGC-1α is multi-faceted with speculation as to whether this protein is a key transducer of external stimuli, in particular when cellular stress is occurring [69]. In the context of exercise several factors have been implicated in the regulation of PGC-1α including AMPK, SIRT1, p38 MAPK and calcium signalling via the myocyte-specific enhancer factor 2C (MEF2C) and D (MEF2D), cAMP response element-binding protein (CREB) and calcium-dependent protein kinase (CAMK) [69,70,71,72,73,74,75].

**Figure 2:**
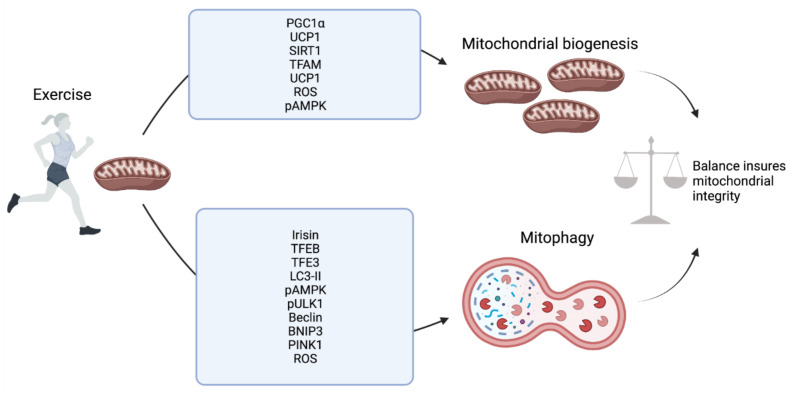
Exercise-mediated regulation of mitochondrial biogenesis and mitophagy at the molecular level.

Autophagy, mitophagy and mitochondrial biogenesis must be carefully regulated so as to maintain a balance of removing damaged organelles and replenishing with new organelles and mitochondria [73,76,77]. Disruption or dysfunction of this balance can lead to the diminished capacity for positive adaption in response to exercise. In serious cases, smaladaptive mitochondrial homeostasis may reduce the capacity to respond to exercise at all. This has been observed in the skeletal muscle tissue of patients affected with autophagy, mitophagy or mitochondrial biogenesis disorders and in the genetic models where these pathways are affected. These individuals are unable to provide the metabolic adaptions required to maintain exercise throughout the body. In the following sections, we will discuss the adaptive measures and specific pathways involved in response to exercise in a variety of cell and tissue types (Figure 2).

When considering mitochondrial dynamics, it is important to consider the role of regulators of mitochondrial cristae remodelling. Cristae structure of the mitochondria influences the respiratory function of cells, whereby genetic and apoptotic alterations of cristae structure negatively affect the cristae structure assembly and activity of respiratory chain complexes in both in vitro and in vivo systems. The ultrastructure and regulation of cristae shape is dependent upon so-called ‘mitochondria-shaping’ proteins. Such proteins include Mitofusions (MFN) 1 and 2 which orchestrate organellar fusion. Specifically, MFN1 cooperates with protein optic atrophy 1 (OPA1), a dynamin-related protein, whereas MFN2 has additional functions of tethering the endoplasmic reticulum and mitochondria. In addition, the fission of mitochondria is influenced by cytoplasmic dynamin-related protein 1 which translocate to the mitochondria following a calcineurin-dependent dephosphorylation regulation. The regulation of cristae remodelling and cristae shape is critical for the assembly of stable respiratory chain complexes into super complex structures that facilitate increased electron flow channeling during respiration [76,78]. As such, stabilisation of respiratory chain complexes affects the mitochondrial respiratory efficiency. Exercise has been demonstrated to impact the stoichiometry of the SC formation, whereby there is a shift towards functional SC formation after training, coupled with increased muscle respiration of humans [77]. Such findings indicate the ‘plasticity’ model of SC formation, whereby free and super-assembled complexes exist and can be influenced to form by changes in energy demand. This research area is developing. Currently, there is limited evidence to demonstrate whether alterations to SC assembly is critical in regulating exercise-mediated benefits. Continued research in this field will illuminate the importance and translational potential of manipulating SCs to improve functional and physiological outcomes of exercise training.

## 2. Skeletal Muscle

Human skeletal muscle tissue makes up a major part of weight in lean healthy individuals [5,79]. Anatomically, this tissue type is arranged in bundles of multinucleated fibers that can be categorised as either slow (type I) or fast (type IIa, x/d and b) as well as being categorised as either oxidative (types I and IIa) or glycolytic (types II x/d and b). This categorisation depends on the contraction rate, type of myosin heavy chain gene expressed and the energy source used, either aerobic (for oxidative) or glycolysis (for glycolytic) fibers, tissue [5,80]. In addition, the number of mitochondria differs between the fiber types. The oxidative fibers typically have a relatively far greater number of mitochondria than glycolytic fiber counterparts [5,80]. These mitochondria have been shown to exist in distinct cellular compartments, classically subsarcolemmally (SS) or intermyofibrillarly (IFM) as well as the more recently described paravascular, I-band, fiber parallel and cross fiber connection mitochondria. These mitochondria in various subcellular locations work in concert to meet the energy demands of muscle contraction [5,81]. In addition to these muscle fibers, muscle stem cells, termed satellite cells, are also present in the tissue and act to regenerate and replenish damaged fibers through differentiation. Given the high-energy demands put upon skeletal muscle during exercise, it is unsurprisingly that this tissue is highly plastic in nature adapting to periods of use and inactivity quickly. A growing consensus is emerging that supports that autophagy, mitophagy and mitochondrial biogenesis being key to this adaptability. A more comprehensive understanding of the molecular pathways surrounding this is key to understanding exercised induced adaptions.

The first description of autophagy in response to exercise came in 1984 when Salminen et al. noted that mice that had undergone 9 h of strenuous treadmill running developed an increased number of vacuoles that were also increased in size [82]. However, it was not until over twenty-five years later that the first studies examining the molecular pathways involved in the induction of autophagy in response to exercise would be performed. The first of these, by Grumati et al. in 2011, found that acute treadmill exercise in WT mice (1 h of running with progressively increasing speed) was able to induce increased LC3I-to-LC3II conversion. However, in COL6A knockout mice (a model where autophagy is impaired) they found these mice had diminished capacity for exercise and actually exercise stress in the absence of autophagy caused damage to the skeletal muscle tissue [83]. The necessity for autophagy has been confirmed in several subsequent studies including a study where acute treadmill exercise in mice for just 15 min was able to induce an increase in autophagy, identifying that posttranslational modification of mTORC1 or AMPK plays an initial role in this process [83,84]. This early onset of autophagy appears to be a vital response for maintaining cellular homeostasis and clearing damaged organelles during exercise [42,83]. However, a short-term response is not the only one to be seen in skeletal muscle. Long-term adaptive responses are also stimulated, through transcription factor-induced gene expression, which prime the skeletal muscle for future bouts of exercise. This includes FOXO3 and FOXO1, TFEB and TFE3 and the mitochondrial biogenesis regulator PGC-1α [15,16,34,35,85,86,87,88,89]. Both FOXO3 and FOXO1 have been shown to be induced in response to increased AMPK, SIRT1 and p38 MAPK which in themselves all show increased activity after exercise [37,39,40,90,91,92]. Following activation, both FOXO3 and FOXO1 induce the expression of a host of critical ATG’s such as LC3, FOXO1 also has direct effects inducing autophagy [35,38,88,93,94]. In addition, FOXO3 is classically under the control of the Akt pathway in skeletal muscle, Akt shows reduced activity during exercise, and this relates to an increase in FOXO3 nuclear translocation [35,85]. This is proposed to act through a reduction in mTORC1 activity however, studies investigating mTORC1 inhibition in unexercised skeletal muscle find only a 10% reduction in autophagy compared to a 50% reduction when inhibiting Akt, indicating other factors may be more important in this process [35,41,42,88,95]. In relation to this, TFEB and TFE3, which are both strongly influenced by mTORC1 signalling in other tissues, show increased nuclear localisation in response to exercise. Furthermore, when TFEB and TFE3 are knocked out in mouse models the capacity for exercise is diminished [34,89,96]. The degree of importance of mTORC1 signalling in skeletal muscle autophagy is questionable, indicating that other factors may be involved in the activity of these two transcription factors. This could include Ca^2+^ signalling with evidence to support the phosphatase calcineurin dephosphorylating TFEB in response to increased intracellular Ca^2+,^ as is seen in skeletal muscle during exercise [97]. Additionally, both AMPK and PGC-1α have been implicated in the activity of TFEB specifically. When phosphorylated, AMPK is known to inhibit mTORC1 activity that would allow for TFEB and TFE3 nuclear localisation [93,94]. AMPK may have direct effects on the phosphorylation status of TFEB, independent of mTORC1, with evidence supporting this in zebrafish skeletal muscle studies. Further research is required to fully understand this dynamic especially in the context of exercise [95]. PGC-1α has also been implicated in the control of not only TFEB and TFE3 but also other autophagy-related proteins including LC3 and BNIP3 [15,40,96,98]. However, there is a degree of ambiguity in this as research also indicates that TFEB can induce PGC-1α upregulation in the liver and FOXO1 in adipocytes suggesting a role of crosstalk in the long-term autophagy-related response [96,99].

As a form of autophagy itself, mitophagy also has been shown to increase during exercise, including in Salminen et al.’s initial observations where increased mitochondrial engulfment into lysosomes was described [82]. In agreement with this, a number of studies have shown the inhibition or attenuation of autophagy leads to an accumulation of damaged and dysfunctional mitochondria that are responsible for diminished exercise capacity and adaptive responses [34,46,76,86,89,91,96]. Despite this, a full molecular understanding of mitophagy in response to exercise in skeletal muscle is still to be achieved. On top of the previously mentioned factors, involved in overall autophagy induction in response to exercise, there is evidence to support mitophagy-specific changes. This includes in vivo increases in mitochondrial targeting for mitophagy in response to acute bouts of exercise as well as increased expression of BNIP3 consistently shown in both acute bouts of aerobic exercise and in trained animals [46,76,91,100]. A study utilising PARKIN knockout mice likewise illustrated increased expression of PARKIN in wild type mice both after an initial acute bout of aerobic exercise but also increased expression is observed at basal levels in trained mice (trained for 6 weeks with voluntary wheel running) [101]. It was also shown that PARKIN is localised to the mitochondrial membrane and primed for mitophagy induction in response to exercise [101]. This study concluded that PARKIN is essential for mitophagy flux in response to exercise and is essential for maintaining basal mitochondrial function [101]. Both AMPK and PGC-1α have been implicated in the upstream control of mitophagy in response to acute bouts of exercise in skeletal muscle [46,76,91,102]. Laker et al., provided the first direct evidence that phosphorylated AMPK (in response to 90 min of treadmill running) is indispensable in the initial induction of mitophagy [46]. Utilising a reporter gene system, named *pMitoTimer*, it was established that in wild type mice mitophagy was induced in response to this exercise challenge at 6 h post-exercise and this was not observed in AMPK dominant-negative transgenic mice [46]. Recently, investigation of AMPKs induction of mitophagy in C2C12 cells indicated that this may be independent of the classical PINK1-Parkin pathway for mitophagy induction [103]. Interestingly, it has also been demonstrated that PINK1 may not be fully necessary for the induction of mitophagy in vivo at basal levels, especially in a number of key metabolically active tissues such as the liver and pancreas [104]. However, it should be noted that in muscle tissues obtained from the PINK1 knockout mice used in this paper did indicate a small, but consistent, increase in mitochondrial content, especially in more glycolytic muscle groups. This indicates a variability in the necessity of PINK1 and possibly other mitophagy machinery in a metabolically hinged context [104]. Given this, it would be interesting to establish the necessity for not only PINK1 but also AMPK in the induction of exercise-induced mitophagy. Equally, PGC-1α is strongly implicated in the control and induction of mitophagy in the response to exercise. PGC-1α knockout mice show ablated mitophagy induction through reduced LC3II and PARKIN mitochondrial localisation, in response to exercise as well as a decline in exercise performance in these animals being observed [89,98]. Interestingly, despite acute exercise inducing mitophagy within 6 h and causing an increase in mitophagy related proteins, repetitive training appears to result in a decrease in mitophagic flux [76,77,102,105]. Although a clear explanation for this remains to be seen, it has been suggested that this may be because the overall capacity and effectiveness of mitochondria are improved in response to repeated bouts of exercise reducing the need for mitophagy to remove damaged mitochondria [106,107]. However, as the mitophagy machinery is increased, it is possible that the muscle tissue is remaining ‘primed’ for mitochondrial clearance in response to a sufficient exercise challenge. It could additionally be the case that the relatively small number of publications investigating mitophagy in response to exercise in trained individuals are doing so at either an insufficient exercise intensity or at sub-optimal time point(s). Indeed, it was observed in the original observations in the 1980s that the peak for mitochondrial engulfment into lysosomes was 72 h after exercise, whereas studies currently have not exceeded 24 h before isolating tissue samples. It may be the case that, after a period of repetitive training, the expected timeframe for mitophagy induction is shifted. Further investigation is required to understand the importance of mitophagy in exercise, especially following training.

**Figure 3:**
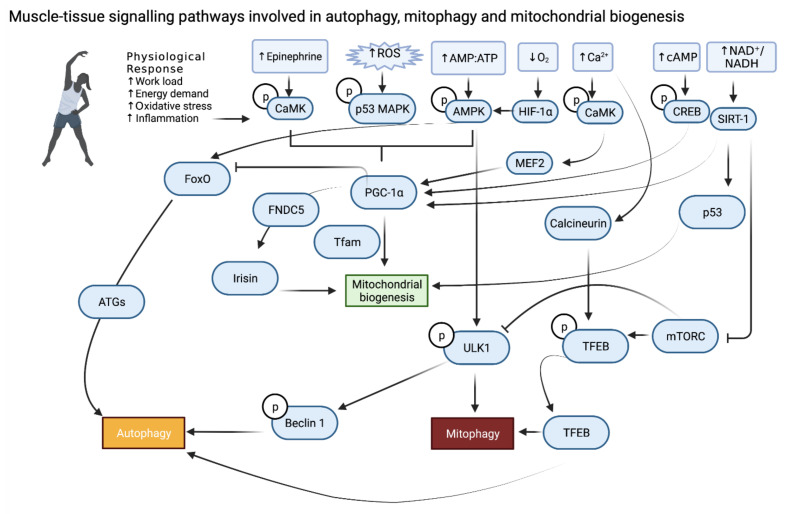
Exercise-induced muscle tissue specific molecular signalling pathways involved in autophagy, mitophagy and mitochondrial biogenesis.

It is also important to understand mitochondrial biogenesis in skeletal muscle in response to exercise. As mentioned, PGC-1α is the master regulator of mitochondrial biogenesis and is regulated by several factors activated during exercise. In skeletal muscle, in response to both acute exercise and exercise training, PGC-1α expression at both the mRNA and protein level increases in an exercise intensity-dependent manner [15,88,100,102,108,109]. Likewise, PGC-1α activity and translocation to the nucleus increases during exercise in skeletal muscle with a significant increase in nuclear PGC-1α detected 3 h after high-intensity training in human male subjects, with this returning to basal levels after 24 h [110]. In concurrence with this, mRNA levels of PGC-1α, as well as some downstream mitochondrial genes such as cytochrome c oxidase II and IV subunits (COXII and COXIV), significantly increased at 3 h with a subsequent increase in protein level observed at 24 h [110]. Following PGC-1α nuclear translocation, along with several other important promoters, PGC-1α has been shown to induce nuclear respiratory factor 1 and 2 (NRF1 and 2). Collectively, NRF1 and NRF2 have been shown to play an important role in the nuclear gene expression of various mitochondrial and respiration related proteins with NRF1 shown to induce TFAM induction in a PGC-1α dependent manner in C2C12 myoblasts and myotubes and collectively [69,111,112]. TFAM, as mentioned, then plays a critical role in inducing mitochondrial DNA transcription and consequent mitochondrial biogenesis [105]. However, it should be noted that in skeletal muscle, although a degree of consensus exists that these proteins are induced in response to exercise and play a role in the biogenesis process, the degree, type and length of single bout or exercise training required for their induction is not well-established [113]. Despite this, there is an emerging and increasingly clear description of the key molecular mechanisms underpinning exercise-dependent effects on mitophagy, autophagy and mitochondrial biogenesis in muscle (Figure 3).

## 3. Liver

The liver is critical in regulating circulating blood glucose levels during times of exercise, or energy deprivation (e.g., fasting state). The mitochondria present in hepatic cells are responsible for fuelling the gluconeogenic event, whereby fatty acids are lipolyzed from hepatic tissue to form ATP [114]. In comparison to non-exercised counterparts, rats that have undergone eight weeks of running on treadmills have increased activity of mitochondrial complexes I, IV, and V indicative of mitochondrial biogenesis [115]. Voluntary exercise, in the form of wheel running, is also demonstrated to increase hepatic mitochondrial content and function in specific rat models [116,117]. Such studies support the notion that exercise enhances hepatic mitochondrial function, mediated by mitochondrial biogenesis. However, the specific molecular mechanisms which mediated mitochondrial homeostasis in response to exercise in the liver requires further investigation.

Hepatic autophagy is mediated by exercise training in an acute and sustained manner. Indeed, it has previously been shown that even a single exercise event can regulate autophagy in the liver [84]. There is emerging evidence that PGC-1α is a molecular player in the regulation of exercise-dependent adaptations in liver. This transcriptional coactivator increases in mouse liver following acute exercise events [118,111]. However, alternate findings indicating a PGC-1α-independent regulation of hepatic autophagy and mitophagy in response to exercise and as such this mechanism requires further confirmatory investigation [111].

The liver is critical in regulating lipid homeostasis. Excess fat and alcohol intake can result in pathological increases in the lipid content of the liver and may result in the development of NASH or NAFLD. These diseases have a significant morbidity and mortality burden on the global population [112]. Exercise is a promising tool to address fatty liver disease, and this is thought to be due to the enhancement of autophagy processes [84,119]. Mitophagy selectively clears the dysfunctional mitochondria present in the liver to prevent hepatic bioenergetic failure and abrogated lipid metabolism [120]. This process may be mediated through exercise, and importantly in muscle-liver cross-talk, independent of diet modification.Skeletal muscle is capable of secreting a number of factors which are collectively termed the ‘myokines’ (including, for example, hormones, chemokines, growth factors and cytokines). One such muscle-released myokine is C1q-TNF-related protein 5 (CTPR5) which promotes glucose uptake and fatty acid oxidation. Humans who undergo aerobic exercise have reduced levels of CRTP5, whilst high-fat diet-fed mice that are CRTP5-null present with reduced hepatic steatosis. The reduction in CRTP5 after exercise inhibits the mTORC1 complex, which in turn enhances autophagy that may mediate the abnormal mitochondrial clearance in liver cells [121]. An alternate myokine that has also received attention is irisin. This exercise-induced myokine has been shown to induce AMPK signalling and this would cause a subsequent reduction in hepatic cell triglyceride accumulation [122]. As such, it is postulated that muscle-derived irisin circulates and causes autophagy stimulation in the hepatic cells. There is wide debate surrounding the role of irisin, with controversy surrounding the determined increase in irisin following exercise. One study report, through tandem mass spectrometry analysis, that high-intensity exercise resulted in a 19% increase in circulating irisin [123]. However, this study assessed only 10 individuals, and as such confidence in the findings is limited.

Exercise and caloric restriction share parallels in which they both extend lifespan and have certain physiological benefits. It is proposed that caloric restriction mediated benefits are due to the induction of autophagy [124]. Caloric restriction leads to the stimulation of AMPK, due to nutrient deficiency and alterations to the ATP/ADP ratio. This, in turn, suppresses mTORC1 and leads to ULK1 activation [124]. This pathway is upstream of autophagy and may be the causative mechanism of caloric-restriction induced autophagy in the liver.

There is emerging evidence suggesting that training intensity itself can have differing effects on modulating autophagy in the liver. Differing intensities of exercise result in varying preferences for the main fuel source. For example, lower intensity exercise is fuelled mainly by lipids, whereas higher intensity exercise leads to glucose as the preferred fuel source [125,126,127,128]. The utilisation of lipids for an energy source is beneficial in preventing excessive accumulation of lipids within the hepatocytes, a phenomenon that is also mediated by changes in regulatory autophagy processes. Wistar rats that have undergone different intensity training exercise including low intensity (10m/min for 30 min) moderate intensity (20 m/min for 30 min) and high intensity (30 m/min for 30 min), 5 days per week for a total of 8 weeks, with non-training (sedentary) rats acting as control [125]. This study identified an increase in hepatic protein levels of Beclin-1, ATG5, LC3 in moderate and high intensity exercised rats compared to controls, indicative of increased autophagy processes [125]. Beclin-1 is identified as a major autophagy initiating protein, responsible for initiating the BECN-1-ATG14-vacuolar sorting protein 34-VPS15 class III P23K core that is critical for the onset of autophagy [87,129,130]. Concomitantly, moderate- and high-intensity exercised rats exhibited decreased serum triglyceride, indicating that exercise-dependent activation of hepatic autophagy may mediate hepatic lipid metabolism (via lipophagy induction) [125]. This study would be strengthened by the inclusion of electron microscopy to confirm lipophagy and the inclusion of female rats to determine whether sexually dimorphic effects of exercise-induced autophagy and regulation of hepatic liver triglyceride is evident. However, this study supports the concept that different training intensities are associated with varying autophagy and subsequent histopathological findings in the liver [125]. Emerging evidence identifies sex-based differences in the response to exercise in a variety of tissues. For example, decreasing sex-hormones (due to ageing, for example) negatively affects the ability of the cardiovascular system to remodel in a sex-specific manner [131]. Furthermore, substrate metabolism in exercise training has bene shown to exhibit sex-based differences in relation to sex-steroids, in particular with relation to respiratory exchange ratio [129,132,133]. Further research is required to determine the effect of sex-steroid and sexually dimorphic responses at the cellular level in relation to exercise-effects.

An alternate study assessed low-intensity exercise and acute shifts in the liver in male c57BL/6J mice. This involved 1 h treadmill exercise training per day, 5 days per week for a 6-week duration, with sedentary mice used as controls. This revealed a robust and fast induction of hepatic PGC-1α immediately after exercise, although effects diminished over time, returning to basal 3 h after exercise [134]. As discussed, PGC-1α is a major activator of mitochondrial biogenesis and as such improved mitochondrial function/turnover may mediate the beneficial effects of exercise on hepatic function. This is supported by several studies [135,136,137].

By determining the pathways that regulate the adaptive responses to exercise in the liver, it is possible that such pathways may be targeted to address the disease state. One such example is in the case of non-alcoholic fatty liver disease, whereby there is an aberrant accumulation of liver triglycerides, damaged and dysregulated mitochondrial biogenesis. It has been demonstrated that aerobic exercise training can result in favourable outcomes in terms of metabolic health and liver function in ob/ob mice with NAFLD [138]. The exercise-trained mice were found to have significantly increased hepatic *Pgc1α* gene expression indicating enhanced mitochondrial biogenesis alongside other improved metabolic parameters which mediated improved hepatic energetic functionality. Mice that are fed a high-fat diet are associated with increased hepatic triglyceride and disrupted liver metabolism, with many suggesting that high-fat diet changes disturb the regulation of liver autophagy [130,139]. This is due, in part, to the changes in membrane-lipid composition of high-fat diet-fed mice which decreases the autophagic fusion capacity [140]. There is continued debate regarding the role of high-fat diet in relation to promoting or inhibiting autophagy within the liver. For example, several studies show that high-fat diet feeding increases the LC3II/LC3I ratio, increased AMPK phosphorylation and mTORC1 dephosphorylation [141,142,143,144]. On the other hand, alternate studies demonstrate a decrease in LC3II and AMPK signalling along with increased hepatic p62 protein levels which is indicative of inhibited autophagy processes in the liver [145,146,147,148,149]. It is critical that further work be carried out to determine the true effect of high-fat diet feeding on the regulation of autophagy processes in the liver. Despite this, it is well known that exercise training can induce positive effects on hepatic metabolism in high-fat diet feeding scenarios for rodents. It has been shown that exercise training is able to ameliorate the HFD-induced changes in AMPK and mTORC1 phosphorylation, LC3I and LC3II levels and p62 protein levels in the liver [146], and that voluntary wheel running is associated with restoration of mitochondrial quality impairment [150]. However, it is undetermined whether exercise training after a prolonged period of high-fat diet feeding can resolve the diet-induced dysregulated hepatic autophagy and mitophagy and this requires further analysis. Furthermore, PGC-1α has been determined as a major regulator of liver mitochondrial biogenesis, but whether this is true in the context of acute exercise or training-induced hepatic autophagy in high-fat diet-fed mice remains to be determined. One study has aimed to identify whether several weeks of high-fat fructose diet feeding and associated changes in liver mitophagy and autophagy processes can be improved following exercise training, resulting in restored hepatic autophagy regulation. The feeding of a high-fat fructose diet resulted in increased hepatic parkin-BN1P3 dimer protein and altered LC3II/LC3II ratio [111]. Following exercise training, a reversal of the high-fat fructose diet-induced changes to LC3II and LC3I ratio was observed, and exercise was also shown to rescue the diet-induced reduction in *Pgc1α* mRNA expression in the liver, which is in line with findings from others [127,151,152,153]. This work by Dethlefsen et al. indicates that exercise training of high-fat fructose diet fed mice increases the capacity for mitophagy within the liver [111]. The modern lifestyle, coupled with physical inactivity and dietary excess, is bearing witness to increased incidence of fatty liver disorders and altered liver mitochondrial function. Exercise, and the exercise-induced molecular mechanisms, may hold the key to improving mitochondrial homeostasis, health and quality and represents a critical research field. Many questions remain unanswered within this field and continued investigative efforts are warranted to advance the field at the basic and translational level.

## 4. Adipose

Adipose tissue has a high degree of mitochondrial plasticity which facilitates its ability to handle flux in energy demand and to handle excess lipids [151,154]. When mitochondrial health is impaired pathological adipose tissue function is observed, which results in increased cytosolic free fatty acids, aberrant glucose uptake by adipose cells and increased triglyceride synthesis [152,155]. This adipose cell malfunctioning, and resultant poor storage of fat, results in an increased inflammatory profile of the cells, and higher production of reactive oxygen species [153,156,157]. This in turn damages other mitochondria within the cell and worsens mitochondrial functionality [158]. As such, quality regulation of mitochondrial function is critical to facilitate the physiological function of adipose tissue and dynamic metabolic adaptations to exercise.

Adipose tissue can be split into two distinct categories, white adipose tissue (WAT) and brown adipose tissue (BAT). WAT functions to store lipids in times of caloric excess, which can subsequently be used as a fuel source in times of caloric deficit. BAT represents a specialised thermogenic organ that, following cold stimulation, metabolises nutrients (such as glucose and fatty acids) to generate heat and maintain body temperature [159,160]. This unique function of BAT is facilitated by the high abundance of mitochondria which are key to enabling the maintenance of homeothermy. Within BAT, there is a proton motive potential across the inner membrane of the mitochondria. This is then directly converted to heat by the function of the uncoupling protein 1 (UCP1)-mediated proton leak. Adult humans, and rodents, also have so-called ‘beige adipocytes’, which are inducible, brown-like adipocytes present within WAT [161,162]. These can be influenced to form by exposure to various environmental or pharmacological stimuli (e.g., cold exposure, norepinephrine exposure, exercise), and express relatively higher levels of UCP1 and mitochondrial content in comparison to classical WAT. Treatments that can enhance mitochondrial biogenesis, and initial studies revealed that diabetic rodents and overweight/abuse humans exhibit insulin resistance coupled with reduced mitochondrial functionality and content in their WAT [163,164]. Given that exercise-training leads to the reduction in adipose tissue mass, and favourable physiological results are observed when adipose mitochondrial quantity and quality is maintained, it is plausible that beneficial exercise adaptations in adipose tissue are mediated through mitochondrial regulation.

One important role of adipose tissue is to facilitate the release of stored fatty acids into the circulation during times of energy demands, such as exercise. The released fatty acids are subsequently taken up and oxidised by highly metabolic tissues. After 30 min of moderate exercise, the lipolysis rate throughout whole-body adipose tissue is increased 2–3 fold in comparison to resting rates, and up to 5-fold after 4 h [165,166] Exercise has been demonstrated to enhance mitochondrial biogenesis in the WAT [167]. Putative findings demonstrate that PGC-1α is a key regulator of mitochondrial biogenesis in adipose tissue, allowing adaptation to meet the increase in energy demand during acute exercise. Indeed, it is shown that PGC-1α levels increased after an acute endurance exercise activity [15]. An acute exercise of 90 min in PGC-1α knockout mice revealed a decrease by 40% of mitochondrial content accompanied by a 25% decrease in running performance and significant acidosis compared to control mice [89]. Furthermore, this exercise training resulted in enhanced autophagic and mitophagic flux in WT mice, with this effect not observed in PGC-1α KO mice [89]. Such findings indicate a role of PGC-1α in coordinating the increased mitochondrial turnover as an effect of acute exercise. Rats that exercised for 4 weeks, with 2 h of daily swim training, exhibit increased mitochondrial marker proteins and *Pgc1α* mRNA expression in WAT (specifically, epididymal and retroperitoneal fat depots), coupled with increased markers of mitochondrial biogenesis including CORE1, COXIV and citrate synthase activity [167]. A similar finding was observed after an acute exercise training of 2 h, although increased protein content of PGC-1α in WAT was not confirmed in either acute or long-term exercise events [167]. Furthermore, the acute overexpression of PGC-1α in adipose tissue is demonstrated to enhance mitochondrial biogenesis [168]. It is posited that adrenaline attenuates the exercise-dependent increase in *Pgc1α* mRNA expression in white adipose tissue, thus indicating that adrenaline may be a key mediator of the PGC-1α expression induction post-exercise [167]. The specific regulators of PGC-1α in response to exercise in adipose tissue remain to be fully elucidated. Research efforts are required to determine the role of extra-cellular signals (e.g., hormones) on PGC-1α and downstream mitochondrial biogenesis events.

There is evidence to indicate the one important exercise-induced adaptation in WAT involves its beiging/browning. Exercise itself has been shown to induce upregulation of adipose browning genes as *Prdm16* and *Ucp1* in inguinal and sub-cutaneous WAT of rodents, resulting in elevated levels of adipocytes with multilocular lipid droplets (indicative of browning) [169,170,171,172]. This is supported by alternate studies demonstrating that exercise-stimuli induces *Ucp1*-expressing brown-like adipocytes to develop amongst the WAT depots [173,174]. This indicates that mitophagy may be critical to facilitate the browning process of adipose tissue and increased expression of brown adipose tissue-specific genes as an adaptive physiological response to exercise [175,176]. As such, autophagy may be a therapeutic target for translational medicine, whereby the rate of mitophagy may be regulated to ensure appropriate balance in adipose tissue. Irisin, released from muscle, is identified to improve liver insulin action suggesting that the exercise response activates enzymes critical in exercise-induced hepatic glucose metabolism [177]. There is putative evidence that WAT ‘browning’ induced by physical activity, is mediated by irisin (FNC5), which is a PGC-1α-dependent myokine that promotes thermogenesis and UCP1 expression. Irisin is induced in exercise and results in a significant increase in total body energy expenditure coupled with improved insulin sensitivity, thus recapitulating the metabolic benefits of exercise [178]. Transgenic mice which express increased PGC-1α in muscle exhibit significantly increased *Ucp1* mRNA in visceral and inguinal WAT following 3-weeks of wheel running [178] indicating the importance of tissue cross-talk in mediating the adipose thermogenic response to exercise regulated by PGC-1α in vivo. There is increasing evidence that the beneficial effects of irisin are mediated by autophagy. For example, FNDC5 knockout mice demonstrate reduced liver autophagy and fatty acid oxidation. Furthermore, primary hepatocytes isolated from the FNDC5^−/−^ mice exhibit decreased autophagy induction and AMPK activity. This is rescued by the treatment of the AMPK activator AICAR, recovering the autophagy rate. Complementary to this, an overexpression of FNDC5 resulted in resistance to autophagy impairment in hepatic cells. As such, FNDC5 deficiency acts in an AMPK-dependent manner to impair autophagy in hepatic cells and is critical in regulating autophagy events. Whether this is the mechanism at play in adipose tissue remains to be determined. As such, irisin appears capable of inducing selective aspects of the exercise-induced programme in adipose tissue, although further work is required to delineate the precise molecular mechanisms of action in an exercise-specific context, with findings still leading to controversy within the field [179]. The exogenous administration of irisin is identified as a strong therapeutic candidate for disease in which no effective treatment exists, or whereby the condition prohibits exercise.

Although adipose tissue is not a mechanical working tissue during exercise, it has the capacity to oxidise fuel substrates to allow the increased demands for energy to be met during exercise. The physiological adaptations which occur due to exercise are many and varied, with one of the significant events being the increase in eNOS gene expression. This in turn leads to an increase in the production of nitric oxide (NO) by various tissues, which has been shown to promote mitochondrial biogenesis in skeletal and cardiac muscle [180,181,182]. However, the role of NO in adipose tissue, and its potential role in metabolic adaptations to exercise, remained unexplored until recently. A study by Trevellin et al. revealed that exercise training induces mitochondrial biogenesis in the subcutaneous depot of WAT specifically and that this occurs in an eNOS-dependent manner [170]. This was determined using eNOS knockout mice which were swim trained and assessed. This indicated an increase in mitochondrial biogenesis and mitochondrial DNA content in the wild type mice, with an absence of effect in the eNOS mice. The evidence of increased mitochondrial biogenesis included increases in mtDNA content (indicative of mitochondrial mass) and the increase in mitochondrial associated genes such as *Pgc1α*, *Nrf1*, *Tfam* and *CoxIV*. This suggests that eNOS is critical for metabolic adaptation of subcutaneous adipose tissue to exercise training [170]. Whether this is true of other WAT depots (e.g., the gonadal, mesenteric) remains undetermined.

Given the evidence in both muscle and liver of TFEB and TFE3’s effect on energy metabolism, there is a necessity to also investigate the role these proteins have in adipose tissue. Recently, there has been growing evidence to support a role for TFEB in the metabolic adaption to fat under various stimuli. To date, no adipose tissue-specific KO model of TFEB has been generated. However, there is sufficient evidence to indicate an important role for this factor in this tissue. In the 3T3-L1 pre-adipose cell line, differentiation into adipocytes resulted in a progressive increase in TFEB expression and siRNA knockdown of TFEB, both at early and late stage of differentiation, indicated a regulatory role over PPARγ2 (a critical factor in the differentiation process of adipocytes) implying an important role in the differentiation process of these cells [183,184]. Furthermore, an overexpression mouse model of TFEB, whereby TFEB-flox mice were crossed with an adiponectin promoter (adipose tissue-specific) controlled CRE mice, led to a protective effect in response to HFD [185]. These mice showed increased leanness (similar to other overexpression models) lower circulating glucose and improved insulin tolerance, however, the effect on glucose homeostasis was found to be secondary to the effect of adiposity so may not be of direct consequence of TFEB overexpression [185]. The improved leanness was shown to be due to a marked decrease in the size of white adipose tissue (WAT) depots but not brown adipose tissue (BAT) which was unchanged in size but did show decreased lipid content [185]. Further examination of this model indicated that WAT browning (where WAT becomes more like BAT) was occurring with a marked increase in the browning marker UCP1 in these mice. This was shown to be independent of changes in autophagic flux and contrasts with a previous report in 3T3-L1 cells where TFEB induction by FOXO1 was shown to increase autophagy and lead to a decrease in UCP1 expression. Interestingly, a recent study has shown that salt inducible kinase 2 (SIK2) is able to induce both autophagic flux and TFEB expression in 3T3-L1 cells but importantly induced autophagic flux prior to TFEB indicating a separate mechanism of control of autophagy in this system [186]. The overexpression effects in these mice were shown to be dependent, once again, on PGC1α with ablation of this protein halting the beneficial effects of TFEB overexpression in HFD mice as well as blocking increased UCP1 expression and browning [185]. Given the growing appreciation of the potential therapeutically beneficial effects of WAT browning, and how this is induced in response to exercise, understanding TFEB’s role in this process could be of importance.

Although no TFEB adipose-specific KO model exists, effects of TFE3 ablation in adipose tissue from the global KO have been shown. This includes an increase in size and lipid content of both WAT and BAT when fed a HFD [34]. Additionally, it was shown that important BAT related genes including *Ucp1* were ablated in TFE3 KO mice indicating a shared role for TFE3 and TFEB in BAT [34]. siRNA knockdown of TFE3 in 3T3-L1 cells has likewise been shown to play a role in adipocyte differentiation being shown to also play a role in PPARγ expression as well as potentially controlling the expression TFEB with these results supported by overexpression analysis in the same model [183]. Given these findings, it is clearly important to further characterize both TFEB and TFE3’s role in all forms of adipose tissue. By doing so we can expand our understanding of how these proteins affect the metabolic adaptions which take place in these tissue in response to different stresses (such as starvation, HFD or exercise) and how these could be manipulated in a therapeutic context.

There is a paucity of detailed knowledge of the role of autophagy, mitophagy and mitochondrial biogenesis in adipose tissue in response to exercise. Continued efforts to elucidate such mechanisms are further complicated by the nature of adipose tissue itself. Adipose tissue is distributed throughout the anatomy in specific depots: emerging evidence suggests that there is adipose depot-specific difference in response to exercise which has functional consequences for physiology. For example, gonadal WAT has been demonstrated to have a higher mitochondrial electron transport system capacity compared to abdominal WAT in humans [187]. Furthermore, following 12 weeks of exercise training, there was increased mitochondrial respiration and coupling in abdominal WAT specifically [187]. This study further revealed a correlation of abdominal fat distribution and insulin sensitivity, which lost significance when normalised to mtDNA. This indicates that the exercise-induced differences are mediated by increased mitochondrial content as opposed to enhanced mitochondrial function [187]. As such, this study indicates differential changes in mitochondrial respiration adaptations between the anatomically differing WAT depots in response to exercise training. It would be of great interest to assess whether there are mitophagy-mediated mechanisms are at play in such adipose depot-specific adaptations to exercise training. Studies such as this are critical to progress the field in a translational context, given that most of the work to date is reliant upon in vitro investigation and utilisation of mouse models, that both present with translational barriers. In addition, studying adipose tissue is intrinsically complicated by the heterogeneous nature of this tissue. Notably, this applies to WAT that undergoes browning, a process which is initiated by exercise (amongst other stimuli). This necessitates careful dissection of mechanisms at play in specific cell types (e.g., UCP1-expressing, and non-UCP1 expressing WAT) within single depots. Such work is aided by the increasingly complex methods of cellular analysis and requires single-cell omics and integrated methodologies of cellular, molecular, pharmacological, and genetic approaches. The continued use of mouse models has identified intrinsic roles of secreted factors, critical in muscle-adipose tissue cross talk, such as irisin. These factors are associated with the regulation of autophagy, however, there is poor documentation of circulating levels of these important players, representing a shortcoming in research unpicking the mechanisms responsible for exercise-induced autophagy in adipose tissue.

Targeting the role of mitochondrial biogenesis in adipose tissue has become increasingly attractive potential therapeutic avenue to combat disease. Progress in this field will be aided by an increased understanding of the mechanisms that govern mitochondrial quality control through the specified process of mitophagy (Table 1). Such knowledge may identify novel therapeutic modalities. This work must include the assessment of the fundamental sex-specific differences in adipose tissue mitochondrial flux. Adipose tissue, at the basic anatomical level, exhibits sex-specific differences in terms of distribution and adiposity, and this may translate to variation between sexes in the beneficial effects of exercise mediated by mitophagy, mitochondrial biogenesis and autophagy in this depot [188,189].

## 5. Cardiovascular

Cardiac function is required to deliver oxygen and nutrients via blood to peripheral organs. At rest, the cardiac output of humans is approximately 5 L/min: this increases to approximately 25 L/min during incremental exercise, increasing blood flow to the skeletal muscles, cardiac muscle, and the brain [207,208]. This acute functionality of the heart is mediated by complex cellular and metabolic systems that facilitate contractility of the heart and maximum efficiency of its pumping action. As such, the heart represents a highly adaptive organ, able to cope with the demands of various energetic states (e.g., stationary or exercise). Significant cardiac adaptability is evident in chronic exercise, as in the case with athletes, whereby the heart remodels to match the increased workload [209,210]. Often referred to as cardiac hypertrophy, this physiological adaptation is achieved by an increase in the size of cardiac myocytes, energy production capacity and mitochondria number [209,210,211]. The adaptations of the heart and wider cardiovascular system are regulated at the cellular and molecular level by upstream signalling pathways are resultant changes in gene/protein expression. This ensures that there is physiological adaptation of the cardiac tissue in response to exercise whilst preventing pathological cardiac growth and facilitating improved patient prognosis following deleterious cardiovascular events [212,213,214]. The pathological cardiac adaptations are observed in situations of chronic hypertension and heart disease, whereby the functionality of the heart (including cardiac output and contractility) declines. This latter cardiovascular development is not related to exercise training and is beyond the scope of this review.

Investigations into the molecular pathways which govern the cardiac adaptations to exercise has led to the identification of several mechanisms of interest. This includes increased insulin sensitivity, adiposity reduction, decreased oxidative stress and increased mitochondrial function and formation. A more recently emerging area of interest is the specialised process of mitophagy in the heart. This pathway was previously demonstrated in striated, skeletal muscle, whereby microautophagy was identified as a critical player in the exercise-mediated conversion of LC3-I to LC3-II [84,215]. It was shown that enhanced LC3-I maturation to LC3-II was identified in rodent myocardium after completion of acute endurance training [84]. This finding demonstrated that the exercise-induced mitophagy processes occurs in both smooth and striated muscle facilitating clearance of damaged/dysfunctional mitochondria. Furthermore, it is determined that exercise induces mitophagic-mediated cardiac protection, and that exercise sustains optimal mitophagy levels in longer-term temporal contexts [216]

The mitophagy process is critical for adaptations that are exercise-mediated/recruited in striated muscle, (e.g., skeletal and cardiac muscle). A critical adaptation is the remodelling of mitochondria which ensures that there is high quality and mitochondrial function [217], with several other non-mitophagic molecular mechanisms existing including protease activation, antioxidant defense and the unfolded protein response. The mitophagy-mediated metabolic improvements are widely believed to be AMPK-dependent, although it remains incompletely understood whether such benefits are due to short-term skeletal muscle metabolism alterations or from wider systemic effects.

There is significant mitochondrial flexibility that occurs during exercise, facilitating metabolic changes due to exercise. TFEB is shown to undergo nuclear translocation during exercise and plays a role in regulating mitochondrial biogenesis that is significantly enhanced due to exercise. In order to facilitate such increased mitochondrial biogenesis, catabolic mitophagic processes are required to remove dysfunctional organelles that are otherwise detrimental to cellular health, and this is posited as one of the major cardioprotective molecular mechanisms. The specific pathways that mediate mitochondrial biogenesis and mitophagy in this context have received increasing research interest. It has been determined that AMPK phosphorylation at tyrosine 172 and AMPK-dependent ULK1 phosphorylation at serine 555 is necessary for targeting of the lysosome to mitochondria [46]. Furthermore, markers of mitophagy (Beclin1, LC3 and BNIP3) are significantly upregulated in rat myocardium throughout acute exercise, with levels returning to basal following 48 h, indicating that mitophagy increases as a response to oxidative stress and inflammation in the myocardium [215]. A further study assessed the effect of sustained (8-week) exercise in the form of swim training in mice and demonstrated significant autophagic flux and activation of mitochondrial fusion and fission events. When such mice were treated with the autophagosomal degradation blocker colchicine, BNIP3 was increased with concomitantly reduced mitochondrial biogenesis. This adds credence to the importance of mitophagy in the context of mitochondrial biogenesis post-exercise training. [218] Evidence of mitophagy mechanisms in humans has also emerged. Human subjects participated in moderate cycling training and revealed enhanced LC31, BNIP3 and PARKIN levels 2 h after training in obtained muscle biopsies [219].

Further questions are also raised regarding whether tissue-specific targeted autophagic inhibition results in mouse models can be recapitulated in general autophagic inhibited/disturbed models. This cell-autonomous, or non-cell-autonomous mechanism remains incompletely understood. To unravel this, muscle-specific tamoxifen-inducible ATG*7* knockout mice were generated by Lo Verso et al. to investigate inhibition of autophagy [220]. This revealed that skeletal muscle autophagy inhibition prior to exercise has a negligible impact on physical performance, AMPK activation or glucose homeostasis [220]. Furthermore, this study revealed the critical role of autophagy to ensure mitochondrial function in muscle contractions which are damaging, demonstrating a sexually dimorphic response [220]. It is important to consider the potential effects of tamoxifen administration alone on the mitophagy phenotypes, as tamoxifen itself induces toxicity, in turn initiating autophagy and so this should be considered carefully in the interpretation of autophagy-mediated phenotypes in inducible mouse models [221]. Further study demonstrates that mitophagy is critical in cardioprotective function in ischaemic/reperfusion injuries and that there is enhanced Bnip3-mediated autophagy in myocardium of rats which were subjected to intermittent running as a form of preconditioning [222,223]. Comparatively, less is understood regarding exercise-mediated autophagic processes in cardiomyocytes than in skeletal muscle. It has been shown that abnormal autophagy rates in cardiomyocytes (either over-active or under-active) can result in cardiovascular disease, and that exercise is able to restore autophagy to a physiological level [84,214,224,225,226,227,228,229]. Specific research questions must be answered to facilitate the development of novel therapeutics for the prevention and management of cardiovascular diseases. Such research will aid in revealing the molecular mechanisms of control and potential of mitophagy and mitochondrial biogenesis as a target to improve cardiovascular health.

This is important to consider this in the context of cardiovascular disease in various contexts. In the case of extensive exercise training, athletes may develop the condition of cardiac hypertrophy, in which there is a significant increase in the size of the cardiac myocytes with the absence of cell division. In this situation, myocyte mitochondria must proliferate within the cell in order to meet the increased energy demand. It is established that to ensure heart health, the mitochondrial machinery of the heart cells must match the energy demands: this fails in the contexts of high work-load associated hypertrophy [230]. In situations of exercise pressure-overload, there is a switch in which mitochondrial mass and activity decline. This is associated with a decrease in the transcriptional activators of fatty acid oxidation and mitochondrial biogenesis regulator factors such as PGC1-α and PPARα [231,232,233]. This pathological hypertrophy, as an adaptation to exercise, results in loss of adequate cardiac energetic production and maladaptive mitochondrial energy metabolism coupled with a metabolic switch from fatty acid oxidation to glucose utilisation. Indeed, the heart typically catabolises fatty acids that provides 90% of the ATP in the non-diseased state [234]. Clinical studies and basic biology demonstrate metabolic inflexibility in the hypertrophic state, with an inability to utilise fatty acids as an energy source [235,236,237]. The hypertrophic heart exhibits increased reactive oxygen species production and dysfunction of the mitochondrial biogenesis as a result [238]. Therefore, there is strong therapeutic potential of targeting mitochondrial biogenesis in the pathological heart remodeling product of intensified training in professional athletes.

## 6. Conclusions and Future Prospective

Exercise is a key tool in the intervention, prevention, and treatment of individuals with metabolic disease, with increasing evidence supporting a role of autophagy, mitophagy and mitochondrial biogenesis in the exercise-induced protective effects. It is increasingly clear that skeletal muscle exhibits a strong circadian profile, with mitochondrial function peaking in the late afternoon. As such, the positive exercise effects on molecular mechanisms and physiology may also be mediated by specific time of day exercise activity. Continued investigation of the timing of exercise and the molecular responses will aid in improving the efficacy of exercise as a therapeutic tool further and will increase understanding of the role of mitophagy, autophagy and mitochondrial biogenesis within this context. Such work necessitates continued integration of animal and human research models, examining the effects of exercise across multiple levels and across lifespans to aid translational models and pharmacological progression. Exercise training is shown to induce autophagy in a wide number of tissues. It has been shown that autophagy can be activated in an exercise-dependent manner in the cerebral cortex of the brain. Treadmill exercise training has demonstrated increased AMPK and SIRT1 activation in rat brain, both factors of which are capable of upregulating autophagy [239,240]. Given that exercise is recommended as an intervention to improve neuronal health, promoting neurogenesis, delayed neurodegenerative disease and decreasing cognitive decline in ageing, it is possible that exercise-induced neural region-specific autophagy may mediate neuroprotective benefits [241]. The precise molecular mechanisms and potential of exercise-mediated autophagic processes in the brain remain incompletely understood, and further work is required to determine these and whether this is mediated through cell-autonomous or non-cell autonomous systemic means. Increased autophagy activity has also been observed in the pancreatic β cells of acute endurance exercised WT mice, with an absence of increased autophagy observed in exercise-stimulated autophagic-deficient mice [84]. Emerging evidence supports the concept of integrated exercise-induced adaptations including several tissues, mediated by so-termed ‘excerkines’ consisting of signalling molecular, hormones and cytokines: the interplay of such exercise and mitophagy/autophagy/mitochondrial biogenesis represents an important area for continued research.

Furthermore, specific research is required to determine the tissue-specific and tissue crosstalk-mediated autophagic response because of various exercise types including acute, chronic, varying intensity (e.g., high versus maximal), and interval training. This will aid in informing optimal recommendations for exercise-mediated benefits. Particular attention needs to be given to the scientific definitions of terminology surrounding the main themes discussed within this paper. A universal acceptance of the criteria for acute, moderate, and chronic exercise in a species-specific manner would significantly enhance the ability to interpret and compare studies in different laboratories and countries. Similarly, a universal acceptance of the determination of mitochondrial biogenesis would be beneficial to avoid confusion and conflicting interpretations within the literature. Furthermore, there is an inherent sex bias in data, particularly those of animal studies, whereby many studies to date exclude the use of female counterparts. It is imperative that female cohorts be included in future studies to delineate sexually dimorphic mechanisms underpinning the molecular interplay of exercise and mitochondrial regulation in various tissues and whole-body responses. Additionally, assessment of autophagy in the context of metabolism and links with exercise has, for the most part, been conducted utilising knock down mouse models. Although these models provide an insight into the links of exercise and autophagy, this is in the specific context of long-term, life-spanning inhibition of autophagic processes [87,238,239]. As such, the role of autophagy (inhibition/activation) in an acute sense is poorly understood. The design of exercised mouse models may also contribute to potentially confounding results. The utilisation of wheel running, or swimming represents the majority of exercise modalities in autophagy-exercise studies [87,214,218,242,243,244]. Although these data provide an insight into the links between general exercise and autophagy regulation, there is an inherent inability to control the exact duration, intensity, and volume of exercise each animal undergoes. As such, this represents a barrier in the scientific communities’ ability to assess the effect of exercise intensity on autophagy-mediated exercise adaptations. Continued investigation of the mitochondrial adaptations and autophagy events will aid the scientific community in reaching a consensus regarding the beneficial effects of exercise, and to further elucidate the complex and multifactorial molecular mechanisms which underpin this. With increasing interest in the development of exercise mimetics, such work is key to determine the intrinsic and key pathways which may be targeted pharmacologically to glean the whole-body, or tissue specific, benefits of exercise training in humans. Development of exercise mimetics may provide an efficient pharmacological and therapeutic option to optimize mitochondrial biogenesis and mitophagy/autophagy processes in individuals suffering from debilitating mitochondrial disease [245,246]. Additionally, exercise mimetic therapeutics may aid in treating the elderly, who have limited ability to conduct physical exercise and suffer from disease associated with features of mitochondrial dysfunction such as sarcopenia and dementia [247]. There is great clinical potential for exercise mimetics, targeting of mitochondrial biogenesis and mitophagy/autophagy and this important field requires further work to strengthen its translational impact.

## Figures and Tables

**Table 1:** Key exercise-dependent molecular mechanisms regulating adipose tissue.

Tissue	Metabolic Mechanism	Effect of Exercise on Metabolic Mechanism	Effect on Physiology	Reference
Adipose	PGC-1α	Increases expression	Enhances mitochondrial biogenesis	[167]
	PGC1-B	Not exercise-induced	Contributes to WAT browning Induces UCP1 expression.	[190]
	Adrenaline	Increases expression	Enhances PGC-1α mRNA expression post-exercise Enhances mitochondrial biogenesis	[167]
	UCP1	Increases expression	Exercise-dependent increase in UCP1 drives WAT browning, regulated by a balance of mitophagy and mitochondrial biogenesis	[191,192,193,194]
	Irisin	Increased release from muscle	Promotes WAT UCP1-mediated thermogenesis Regulates muscle-adipose tissue cross-talk enhancing WAT browning, mediated by PGC-1α. Facilitates autophagy	[178,195]
	TFEB	Increased TFEB expression and nuclear translocation	TFEB induction by FOXO1 increases autophagy decreases UCP1 expression	[184,196]
	SIK2	Not exercise-induced	Induces autophagic flux and TFEB expression	[186]
	SIRT1	Increased activity	Induces deacetylation of PPARγ and PGC-1α and recruits adipose browning coactivators including PRDm16	[197,198,199]
	Norepinepherine	Increased activity	Induces PGC-1α via p38 MAPK activation and subsequent ATF2 activation.	[200]
	Myokine response (IL-6, IL-10, IL1ra)	Increased	Important in anti-inflammatory response which also mediated by mitophagy to regulate inflammatory tone in response to exercise.	[201,202]
	eNOS	Increases response to exercise	Increases mitochondrial biogenesis	[170]
	FGF21	Increases in response to exercise	Increases mitochondrial biogenesis	[203,204]
	Prdm16	Increases in response to exercise	Induces upregulation of thermogenic genes and WAT adipocyte browning	[205]
	ROS	Increases in response to exercise	Induces mitochondrial biogenesis and induces WAT adipocyte browning	[206]
